# A Novel Gene, *OsRLCK191*, Involved in Culm Strength Improving Lodging Resistance in Rice

**DOI:** 10.3390/ijms252212382

**Published:** 2024-11-18

**Authors:** Huilin Chang, Hanjing Sha, Shiwei Gao, Qing Liu, Yuqiang Liu, Cheng Ma, Bowen Shi, Shoujun Nie

**Affiliations:** 1 Suihua Branch of Heilongjiang Academy of Agricultural Sciences, Suihua 152000, China; huilin_chang1991@sina.com (H.C.); nkxshahanjing@163.com (H.S.); gaoshiwei1118@126.com (S.G.); genjos@163.com (Q.L.); 13846789457@163.com (Y.L.); macheng5555@163.com (C.M.); sbw_agri@163.com (B.S.); 2 Key Laboratory of Germplasm Enhancement, Physiology and Ecology of Food Crops in Cold Region, Ministry of Education, Northeast Agricultural University, Harbin 150030, China; 3 Key Laboratory of Soybean Molecular Design Breeding, State Key Laboratory of Black Soils Conservation and Utilization, Northeast Institute of Geography and Agroecology, Chinese Academy of Sciences, Harbin 150081, China

**Keywords:** rice, culm strength, receptor-like cytoplasmic kinase, RNA-seq

## Abstract

Lodging is one of the major problems in rice production. However, few genes that can explain the culm strength within the temperate *japonica* subspecies have been identified. In this study, we identified *OsRLCK191*, which encodes receptor-like cytoplasmic kinase and plays critical roles in culm strength. *OsRLCK191* mutants were produced by the CRISPR-Cas9 DNA-editing system. Compared with wild types (WTs), the bending moment of the whole plant (WP), the bending moment at breaking (BM), and the section modulus (SM) were decreased in *rlck191* significantly. Although there is no significant decrease in the culm length of *rlck191* compared with the WT; in the mutant, except the length of the fourth internode being significantly increased, the lengths of other internodes are significantly shortened. In addition, the yield traits of panicle length, thousand-seed weight, and seed setting rate decreased significantly in *rlck191*. Moreover, RNA-seq experiments were performed at an early stage of rice panicle differentiation in shoot apex. The differentially expressed genes (DEGs) are mainly involved in cell wall biogenesis, cell wall polysaccharide metabolic processes, cellar component biogenesis, and DNA-binding transcription factors. Transcriptome analysis of the cell wall biological process pathways showed that major genes that participated in the cytokinin oxidase/dehydrogenase family, cellulose synthase catalytic subunit genes, and ethylene response factor family transcription factor were related to culm strength. Our research provides an important theoretical basis for analyzing the lodging resistance mechanism and lodging resistance breeding of temperate *japonica*.

## 1. Introduction

Lodging is one of the main factors restricting the high yield and superior quality of rice. Once lodging occurs, the photosynthetic rate decreases due to the deterioration of the canopy structure, thereby inhibiting the transport of nutrients and water. Rice grains that have been in a high-humidity environment for a long time will germinate, resulting in poor grain quality [1]. Since the 1960s, the “Green Revolution” breeders introduced the semi-dwarf gene sd1 into rice cultivars, aiming to reduce lodging risk by making plant height shorter [2,3]. Even though the *sd1* gene contributes to enhancing lodging resistance, it also has negative effects on culm thickness and breaking-type lodging [4]. Rice breeding strategies for semi-dwarf *japonica* applied weak function *sd1* (*sd1-EQ*, *sd1-r*) in northeast China [5] and cultivars have utilized a strong culm to prevent lodging. In recent years, extreme weather has occurred frequently and the probability of typhoons in the northeast of China has increased. The lodging caused by typhoons in the late stage of rice maturity has attracted more and more attention [6]. However, the risk of lodging still increases in some extreme conditions [7,8], and the application of semi-dwarf cultivars would be a novel approach for lowering biomass [4]. In the “post-green revolution” era, breeding endeavors targeting super rice frequently regard increasing biomass as a breakthrough point. Appropriately raising the height of rice plants constitutes one of the crucial approaches to enhancing biological yield. Meanwhile, higher demands are imposed on these varieties with regard to enhancing the culm strength. It is necessary to breed rice cultivars with higher lodging resistance and a thicker and stiffer culm, even though culm strength is a complex trait controlled by multiple genes.

In recent decades, the identification of quantitative trait loci (QTLs) associated with culm strength in rice, which affects lodging resistance under normal or super typhoon weather, has been a fruitful approach for uncovering the mechanical theory of culm strength in genetic breeding [8,9,10,11,12,13]. A QTL (*prl5*) derived from *indica* variety Kasalath is responsible for the increased pushing resistance (PR) of the lower part of rice. Moreover, it has been reported that *prl5* might affect the characteristics of the lower stems to lodging resistance by typhoons [14]. Then, a new locus for lodging resistance in a typhoon (*lrt5*) with the near-isogenic line under the rice Koshihikari genetic background was demonstrated to enhance the rigidity of the upper culm by starch content [13]. *BSUC11*, the QTL related to the bending-type lodging of the upper part of culms, was also identified from Kasalath [11]. Strong culm 2 (*SCM2*) conferring lodging resistance by increasing culm diameter was isolated and mapped from chromosome segment substituted lines (CSSLs) derived from *indica* variety Habataki and temperate *japonica* variety Sasanishiki, which was identical to *ABERRANT PANICLE ORGANIZATION1* (*APO1*) involved in controlling the rachis branching number of panicles [15]. Meanwhile, QTLs of *SCM1* derived from the strong, thick culm variety Habataki was located on chromosome 1, including Gn1a, which controlled the grain number [16]. *SCM3* and *SCM4* have been identified from the back-cross inbred lines derived from the tropical *japonica* variety, Chugoku 117, and temperate *japonica* variety, Koshihikari. The causal gene for *SCM3* is *Fine Culm 1* (*FC1*), which encodes a transcription factor involved in strigolactone signaling, whereas its alleles thicken culms and increase the number of grains per panicle but decrease the number of panicles and have no effect on yield [17,18,19]. Genome-wide association studies (GWASs) are more efficient and more widely useful for investigating the complex traits of agronomic and commercial importance. OsFBA2 was validated as a candidate gene to contribute to lodging resistance in rice by GWASs, and it was observed that Hap3 was the most promising haplotype in the *japonica* subpopulation [20]. *wp2*, *MHZ5*, and *OsDof-23* were the candidate genes affecting both the long axis (LA) and short axis (SA), the PR and breaking resistance (BR), and the LA, SA, and PR, respectively [21]. Most existing discoveries applied a set of genetic populations or a group of rice accessions derived from *indica* and tropical *japonica* or temperate *japonica*. However, few genes that explain the diversity of culm strength within temperate *japonica* subspecies have been identified.

Utilizing the additive effects of culm strength QTLs and conducting the pyramiding of multiple superior alleles is of great significance for realizing the breeding strategy of cultivating strong culms [16]. Therefore, undetected superior alleles within temperate *japonica* subspecies are a promising new target for further enhancing culm strength. In a previous study, it was found that *OsRLCK191* is a candidate gene for culm thickness, and its superior alleles are unutilized in temperate japonica rice and can be used to improve culm strength and lodging resistance in the future [8]. *OsRLCK191* codes receptor-like cytoplasmic kinase (RLCK). In rice, there are 379 RLCKs have been identified by the HMM-based domain structure and phylogenetic relationships, which belong to a super family of receptor-like kinases (RLKs) [22]. Some of the functionally characterized RLCKs from plants have been shown to play roles in development and stress responses, such as the response to hormones, cell differentiation, growth, development, responses to environmental stresses, and pathogen recognition [23]. Most reports demonstrated that RLCKs are pivot signaling points in plant responses to biotic stress [22,24,25,26,27,28]. RLCKs have been shown to regulate salt tolerance in rice [29,30,31,32,33]. At present, the functions directly associated with culm thickness have not been reported for RLCKs. However, it has been reported that some RLCKs that work as negative regulators of brassinosteroid signaling were involved in panicle number regulation [34]. The RLCK-VND6 module was involved in the abscisic acid-mediated regulation of cell growth, which was demonstrated as a fine-tuned secondary cell wall deposition with precise control [35]. Up to now, no morphological performance has investigated the gene *OsRLCK191* for culm thickness. Here, we report an RLCK that is associated with culm strength in rice. Through RNA-seq, our study identifies an unprecedented fine-regulatory biological process that modulates biomass accumulation and adaptive growth, which may be applicable for crop lodging resistance breeding.

## 2. Results

### 2.1. Production of OsRLCK191 Knockdown Mutants Using CRISPR/Cas9 Method

**Figure 1:**
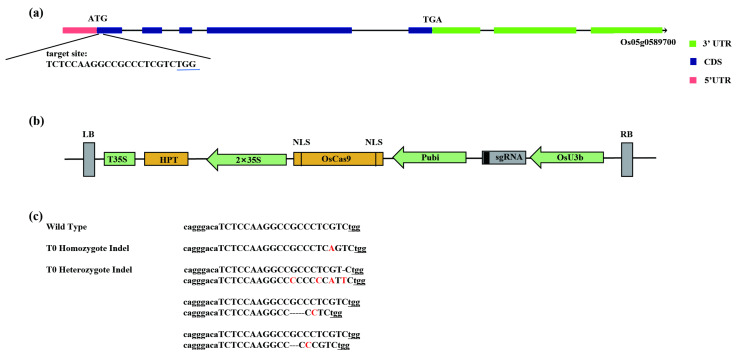
CRISPR/Cas9-induced *OsRLCK191* gene modification in rice. (**a**) A schematic of the *OsRLCK191* gene structure and target site. Introns are indicated with black lines. The protospacer-adjacent motif (PAM) site is underlined; (**b**) A schematic presentation of the T-DNA structure in the CRISPR/Cas9-mediated genome-editing construct. The expression of Cas9 is driven by the maize ubiquitin promoter (Pubi); the expression of the sgRNA scaffold is driven by the rice U3b promoter (OsU6a); the expression of hygromycin (HPT) is driven by two CaMV35S promoters (2 × 35S). Abbreviations: NLS, nuclear localization signal; LB and RB, left border and right border, respectively. (**c**) Nucleotide sequences at the target site in the T_0_ mutant rice plants. The target site nucleotides are indicated with capital letters. The PAM is underlined. The dashes indicate deleted nucleotides. The red letters indicate inserted or substituted nucleotides.

The gene *OsRLCK191* is located on chromosome 5 in rice and related to culm strength as receptor-like cytoplasmic kinase, which consists of five exons and four introns. To verify the function of *OsRLCK191* in rice, the knockdown mutants were constructed by a CRISPR/Cas9 assay. A 20 bp nucleotide sequence in the first exon of *OsRLCK191* (gene ID: LOC_Os05g51190 from Michigan State University’s Rice Genome Annotation Project, RGAP, http://rice.plantbiology.msu.edu/, accessed on 20 January 2020), was chosen as the target site (Figure 1a). The binary plasmid pHUN411-Cas9-OsRLCK191 (Figure 1b) was constructed, which was used to transform rice variety Suijing18 via agrobacterium-mediated transformation. Using site-specific PCR and Sanger sequencing, a total of five mutants were recovered from fourteen T_0_ hygromycin-resistant transgenic plants.

The mutants were detected and then subjected to a zygosity analysis by DNA sequencing PCR products of targets in putative mutants. According to sequencing analyses, homozygous mutations and heterozygous mutations were obtained. T_0_ mutant plants were self-pollinated, and their progenies were genotyped at the target site. We randomly selected T_1_ progenies derived from each T_0_ plant for genotyping analysis. As expected, all of these T_0_ putative homozygotes and their offspring had identical genotypes, suggesting that these homozygous mutant lines could transmit to the next generation stably.

To further obtain rice lines harboring the desired *OsRLCK191* mutations without T-DNA, the mutant individuals were selected without hygromycin resistance. As a result, one type of the homozygous mutants was isolated (Figure 1c) in the T_1_ generation to produce the T_2_ population to identify the culm strength phenotypes.

### 2.2. OsRLCK191-Induced Mutations Involved in Culm Strength

**Figure 2:**
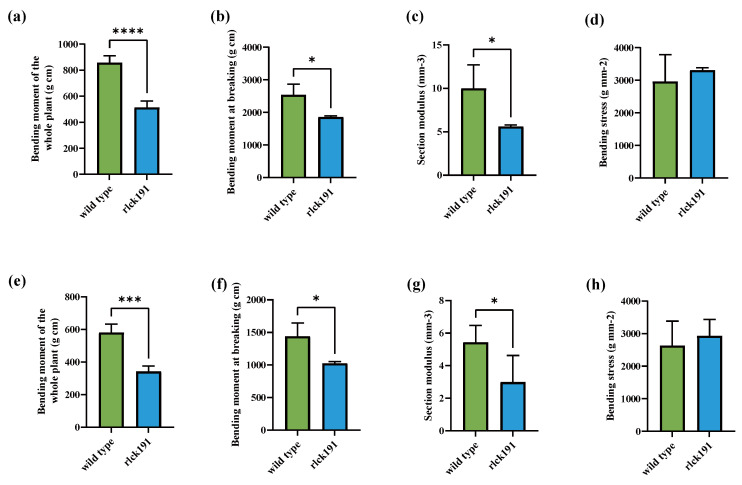
The identification of culm strength in homozygous mutant lines. (**a**–**d**) The bending moment of the whole plant, bending moment at breaking, section modulus, and bending stress of wild-type and mutant lines measured on the second internode. (**e**–**h**) The bending moment of the whole plant, bending moment at breaking, section modulus, and bending stress of wild-type and mutant lines measured on the third internode. Asterisks indicate significant differences between the wild type and mutant: *p* < 0.05 (Tukey’s test). *, ***, and **** represent significant differences at 5%, 1%, and 0.01% levels, respectively.Each bar indicates the mean ± SD (*n* = 6).

**Figure 3:**
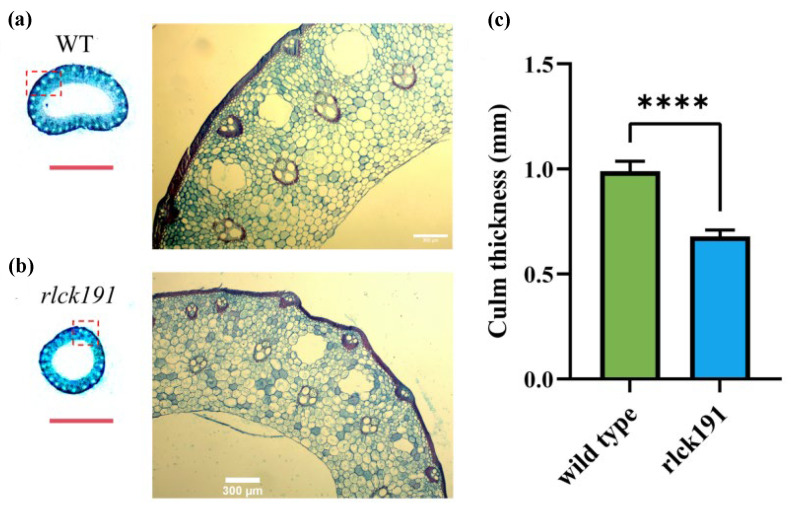
Cross-sections of the basal internode in wild type and *rlck191*. (**a**) Wild type. (**b**) Mutant type *rlck191*. Red dashed box indicates location of amplification. Red scale bars, 0.5 cm; White scale bars, 300 μm. (**c**) Culm thickness of wild type and rlck191. Each bar indicates mean ± SD (*n* = 6), **** represent significant difference at 0.01% level.

To reveal the function of *OsRLCK191* on the traits associated with culm strength for further enhancing lodging resistance, quantitative traits associated with culm strength and agronomic traits of homozygous mutant T_2_ lines and WTs were evaluated. Compared with the second internode of WTs, the bending moment of the whole plant (WP), the bending moment at breaking (BM), and the section modulus (SM) were decreased for 40.03%, 26.87%, and 48.48% significantly, respectively, in *rlck191*, while bending stress (BS) was increased with no significance (Figure 2a–d). In the third internode of *rlck191*, its WP, BM, and SM were 1.77, 1.40, and 1.50 times lower than those of the WT, respectively (Figure 2e–h). For the WT, its higher culm strength depends on the SM, which is directly influenced by its culm diameter and wall thickness, since its culm wall is thicker (Figure 3a–c). On the contrary, the other factor, BS, which is influenced by culm cell wall components such as cellulose, lignin, etc., was not significantly different between the WT and *rlck191*.

### 2.3. Investigation of Agronomic Traits

**Figure 4:**
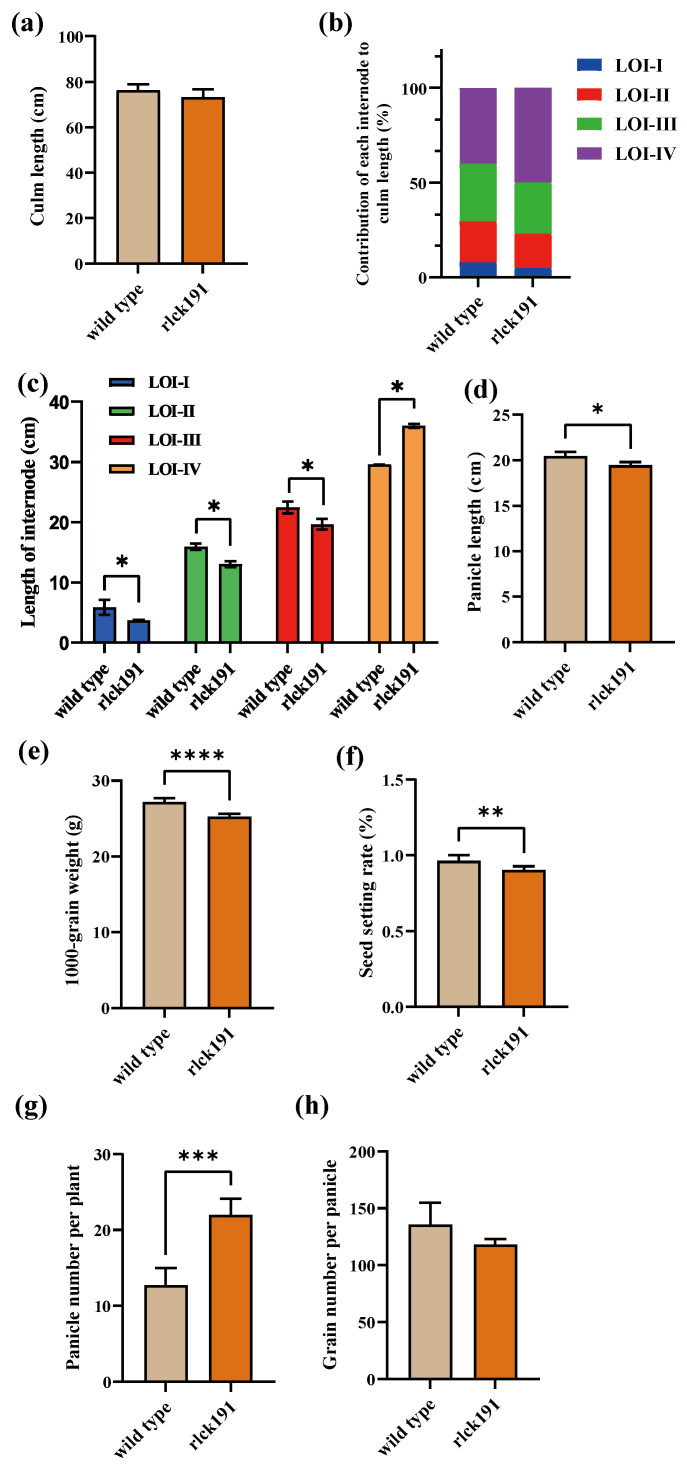
Agronomic trait values of wild type and mutant *rlck191*. (**a**) Culm length. (**b**) Contribution of each internode to plant height. I–V indicate internodes from base to head. (**c**) Length of each internode. Data are expressed as mean ± SD. *, **, ***, and **** represent significant differences at 5%, 1%, 0.1%, and 0.01% levels, respectively. Two-tailed Welch’s *t*-test was performed by Graphpad Prism 9.5 software. (**d**) Panicle length. (**e**) Thousand-grain weight. (**f**) Seed setting rate. (**g**) Panicle number per plant. (**h**) Grain number per panicle.

The plant architecture analysis of the WT and *rlck191* showed that mutant had no change in the plant architecture (Supplementary Figure S1). Although culm length did not change significantly between the WT and *rlck191* (Figure 4a), the variations in each internode were significant; compared with the WT, the length of LOI-I, LOI-II, and LOI-III of *rlck191* were significantly reduced, respectively, while the length of LOI-IV was significantly increased compared with the WT (Figure 4b,c). An analysis of yield-related traits showed that panicle length, thousand-seed weight, and the seed setting rate have significantly decreased in *rlck191* (Figure 4d–f), while the panicle number per plant has increased significantly (Figure 4g). However, the grain number per panicle decreased in *rlck191* with no significance (Figure 4h). Overall, *OsRLCK191* has positive effects on grain yield-related traits and negative effects on the panicle number per plant.

### 2.4. RNA-seq Analysis of OsRLCK191 Mutant in Shoot Apex

To understand the regulatory mechanisms of *OsRLCK191* to affect culm strength and explore new regulatory mechanisms involved in lodging resistance, the shoot apex of Suijing18 and its *rlck191* mutant were studied using RNA-seq at an early stage of rice panicle differentiation. Gene expression was calculated as the fragments per kilobase per million reads (FPKM). For RNA-seq, each library ranged from 40.72 to 52.71 million reads. The clean reads were mapped onto the reference genome via HiSat2 (http://ccb.jhu.edu/software/hisat2/index.shtml, accessed on 9 August 2024) and most of the clean reads (95.68–97.47%) for each library were perfectly mapped to the rice reference genome. FPKM was calculated to measure the expression levels of the transcripts and showed high correlations (Spearman correlation coefficient (SCC) = 0.775–0.978) among the biological replicates. As a result, a total of 66,660 transcripts were detected in at least one sample and 16,685 new transcripts were found compared with the reference genome.

**Figure 5:**
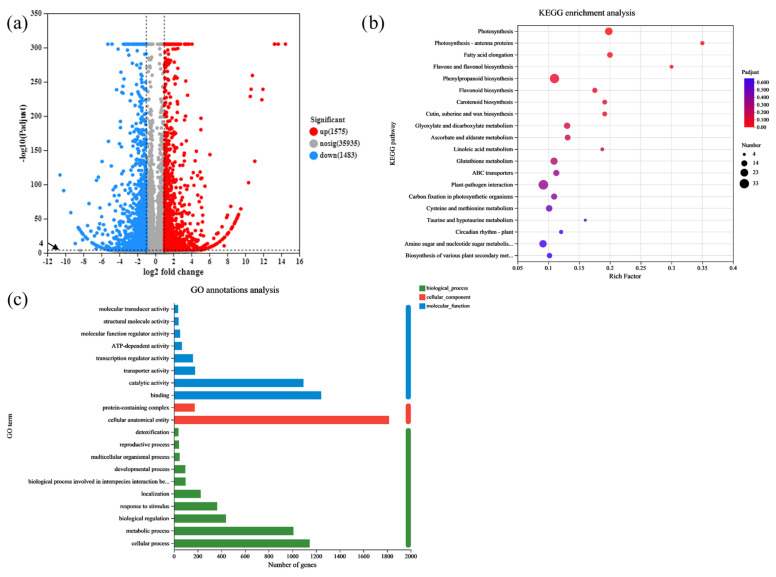
RNA-seq analyses of the *rlck191* mutant. (**a**) The volcano map of DEGs between WT plants and mutant plants. (**b**) The KEGG enrichment analysis of the differentially expressed genes and proteins presented in a bubble chart. (**c**) The GO analysis of the DEGs. Enriched significantly different GO terms with both *p* value < 0.05 and *p* adjust < 0.001.

The differential gene expression of the WT and *rlck191* was examined using DEGseq (https://www.rdocumentation.org/packages/DEGseq/versions/1.26.0, accessed on 9 August 2024) to quantify and analyze the genes. A total of 3058 genes were differentially expressed in shoot apex between the WT and mutant, of which 1483 genes were downregulated and 1575 upregulated (Figure 5a). To determine the biological function of *OsRLCK191*, gene ontology (GO) enrichment was conducted on the DEGs. The GO analysis indicated that the DEGs were separated into 20 different GO terms, including eight terms for molecular function, two terms for cellular components, and 10 terms for biological process (Figure 5c). The Kyoto Encyclopedia of Genes and Genomes (KEGG) pathway enrichment analysis was conducted to further examine the biological pathways responsible for the mechanism of culm strength related to lodging resistance. Among the 20 enriched pathways (Figure 5b) that occurred in the results that were demonstrated to be related to *OsRLCK191*, enriched in special pathways were the MAPK signaling pathway for plants, cysteine and methionine metabolism, plant–pathogen interaction, and plant hormone signal transduction.

**Figure 6:**
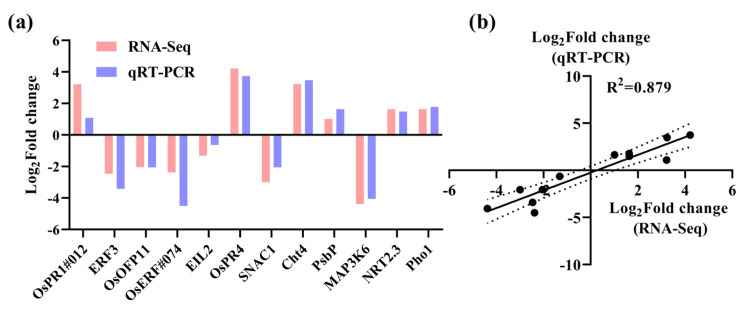
The validation of RNA-seq for the WT and *rlck191*. (**a**) Expression profiles and quantitative levels of DEGs. (**b**) The correlation analysis of qRT-PCR and RNA-seq. The area between the two dotted lines represents the 95% confidence interval.

To confirm the DEGs identified from the transcriptome analysis, qRT-PCR was used to determine the expression levels of DEGs and 12 genes from different GO items that had been carefully selected (Supplementary Table S1). Subsequently, a correlation analysis was implemented. As illustrated in Figure 6, the correlation coefficient R^2^ was determined to be 0.879 between RNA-seq and qRT-PCR, which implies that the sequencing data of RNA-seq possesses a relatively high level of credibility.

**Figure 7:**
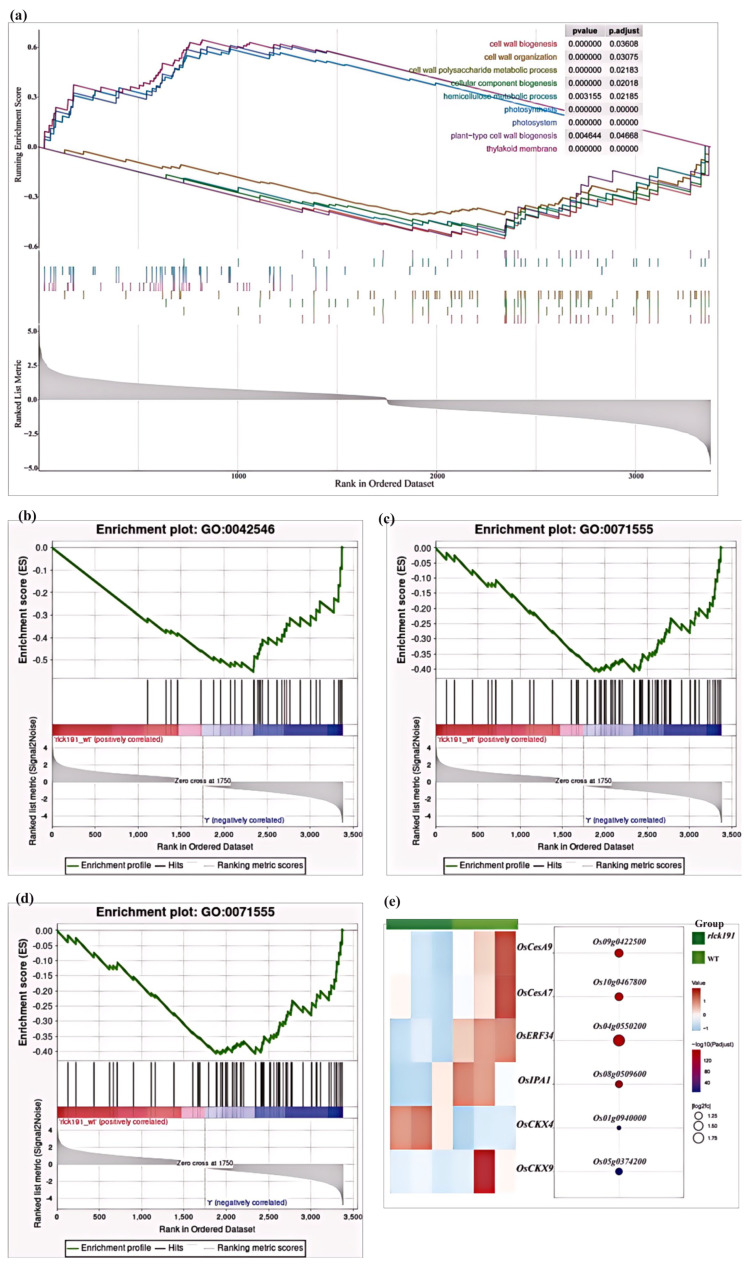
Expression of DEGs between the WT and *rlck191*. (**a**–**d**) The DEGs of the GSEA. (**e**) A heat map of the transcript level of downregulated genes and upregulated genes.

To further confirm that significant genes contain many regulatory factors, we performed a gene set enrichment analysis (GSEA) [36]. We found that the pathway of cell wall biogenesis, cell wall polysaccharide metabolic processes, and cellar component biogenesis were significantly enriched for downregulating in *rlck191* (Figure 7a–d). Moreover, six genes were examined to be related to culm strength, which is responsible for the cytokinin oxidase/dehydrogenase family (*OsCKX4*, *Os01g0940000* and *OsCKX9*, *Os05g0374200*), cellulose synthase catalytic subunit genes (*OsCesA7*, *Os10g0467800* and *OsCesA9*, *Os09g0422500*), ethylene response factor family transcription factor (*OsERF34*, *Os04g0550200*), and squamosa promoter-binding protein-like (*OsSPL14*/*IPA1*, *Os08g0509600*), among which only one gene was upregulated, suggesting upregulation of these pathways (Figure 7e).

## 3. Discussion

To date, RLCKs as kinases in plant play an important role in diverse biological processes, including development, self-incompatibility, response to pathogens and defense, and response to multiple environmental stresses. It has been reported that *OsRLCK191* has been identified as a potentially superior allele for enhancing culm strength but has not been inherited by modern improved cultivars [8]. However, we found that rice varieties bred in Northeast China contain this excellent allele in our previous research using data from MBKbase-Rice [37], such as Suijing 18, Longjing30, Dongnong425, and Hejiang19, indicating that this gene has been utilized to a certain extent in breeding in Northeast China. The BM, WP, SM, and BS are crucial indicators for measuring culm strength. The BM reflects the bending moment generated when the whole plant is subjected to forces. The WP comprehensively takes into account the influence of factors such as plant height, culm morphology, and panicle weight on the degree of culm bending. The SM is related to the shape of the cross-section and the position of the neutral axis. The larger the SM of the cross-section of the culm, the stronger its ability to resist bending deformation, enabling it to better withstand bending stresses from all directions. In this study, the WP and SM decreased substantially in mutant *rlck191*, while BS increased significantly, indicating that *OsRLCK191* has an improvement in culm strength and contributes to lodging resistance.

In contrast to the positive impact of *OsRLCK191* on culm strength and crop yield by increasing the panicle number of per plant, it also exerts negative effects on crop yield by reducing the panicle length, thousand-seed weight, and seed setting rate (Figure 4d–h). Considering the predicted function of these six DEGs selected from the GESA and the phenotypes of the WT and *rlck191*, the overall trend in the expression changes in these genes appears to be reasonable. *OsCKX4* participates in the growth and development of rice roots, basal internodes, tillers, leaves, panicles, and grains. It positively regulates the development of the root system, the diameter of the basal internode, size of flag leaves, panicles, and grains, and negatively regulates tillering [38]. Mutants of *osckx4* and *osckx9* exhibit an increased tiller number, while exhibiting a decreased length of panicles, seed setting rate, and thousand-grain weight [39]. *OsCesA7* and *OsCesA9* are highly expressed in seedlings. The former is not expressed in mature leaves, while the latter is expressed in culm at a mature stage [40,41]. They regulate cell walls in the cortical fiber cells and influence the thickness of culm [42]. *OsERF34* and *RMD* are highly expressed in sclerenchymatous peduncle cells that are fortified by thick secondary cell walls (SCWs), providing mechanical peduncle strength, thus affecting the mechanical strength of the peduncle. On the other hand, *OsERF34* has a positive effect on length of the internode, especially in increasing the length of the top internode. Notably, the length of internodes was significantly decreased in this study, except for the top internode, which might be related to the downregulation of *OsERF34* in the mutant. *IPA1* was identified in GOs and the GSEA (Figure 7a–d). It resolves the tradeoff between the grain number per panicle and tiller number, resulting in a substantial enhancement of grain yield per plant and having an impact on culm strength. Moreover, *IPA1* can be induced to undergo phosphorylation under abiotic stress, which corresponds to the biochemical function of *OsRLCK191* as a kinase and their relationship discovery in the future.

In conclusion, numerous aspects within the research of these genes remain to be further delved into. These genes exert an impact on the growth and development of rice stems, the formation of cell walls, and adaptability to the environment via their individual functions and intricate network regulations among them. For instance, the precise regulatory mechanisms among these genes, particularly the dynamic regulatory associations under diverse environmental circumstances and during different growth and development phases of rice, require further elucidation. Additionally, how to employ these genes in rice molecular breeding to develop rice varieties characterized by high yield, suitable stem bark thickness, high stem strength, and lodging resistance represents a significant avenue for future research. Through further in-depth exploration of the functions and regulatory networks of these genes, it is anticipated that novel theoretical foundations and gene resources will be furnished for high-quality and high-yield rice breeding.

## 4. Materials and Methods

### 4.1. Plant Cultivation

We used *japonica* rice cultivar Suijing18, which have been cultivated with a certain share in Northeast China and carry the superior allele of *OsRLCK191* [8]. The field experiment was conducted at the Suihua branch of the Heilongjiang Academy of Agricultural Sciences (46°36′56″ N, 126°59′17″ E) during April to September in 2022 and 2023. The field planting followed a randomized complete block design with two experimental replications. Each accession was planted in a plot with three rows, with ten plants in each row at a spacing of 16.7 cm × 30 cm. The field management followed the local farmers’ standard practices.

### 4.2. Measurement of Lodging Resistance Traits

At 14 days after heading, five main culms per cultivar were sampled to evaluate the physical parameters related to lodging resistance. After the measurements of the length of five individual internodes (LOI-I, LOI-II, LOI-III, LOI-IV, LOI-V), the bending moment at breaking (BM, g) was measured at a distance of 4 cm from the supporting points by a lodging resistance detector (YYD-1, Zhejiang Top Instruments Co., Ltd., Hangzhou, China). The central section of internodes was sectioned by hand, and the inner and outer diameters a_1_, a_2_, b_1_, and b_2_ were measured using a sliding Vernier caliper, where a1 is the outer diameter of the minor axis, b_1_ is the outer diameter of the major axis, a_2_ is the inner diameter of the minor axis, and b_2_ is the inner diameter of the major axis. The values of traits of lodging resistance were calculated by the following formula according to the method of Ookawa and Ishihara: section modulus (SM, mm^−3^) = π(a_1_^3^b_1_ − a_2_^3^b_2_)/32a_1_; bending stress (BS, g mm^−2^) = BM/SM; bending moment of whole plant (WP, g cm) = the distance from the basal of internode to tip of panicle (SL, cm) × the fresh weight of this section (FW, g); breaking resistance (M, g mm^−2^) = BM × the distance between fulcrums (L, cm); and lodging index (LI) = WP/M.

The other six agronomic traits are culm length (CL), panicle number (PN), panicle length (PL), grain number per panicle (GNP), seed setting rate (SSR), and thousand-grain weight (TGW).

### 4.3. Construction of CRISPR/Cas9 Vector System

According to the experimental requirements, the knockout mutant of *OsRLCK191* (LOC_Os05g51190) was constructed by the CRISPR/Cas9-based genome-editing method. The Cas9 plant expression vector (pHUN411-Cas9) and the sgRNA expression vector (pYLgRNA) were used in this study [43]. For designing gRNA targets, 20 bases upstream of the protospacer adjacent motif (PAM) were selected as candidate target sequences, where NGG is PAM (Figure 1a). A BLAST search (http://blast.ncbi.nlm.nih.gov/Blast.cgi, accessed on 30 July 2024) of the target sequences (including PAM) against the rice genome was conducted to confirm their targeting specificity in the genome. The target sequence has a difference of at least two bases compared with similar non-target sequences within the PAM region. The sgRNAs with a target sequence of *OsRLCK191* were assembled into the vector pHUN411-sg2.0 with flanking primers containing BamHI restriction sites that generated pHUN411-sgRNA-rlck191 (Supplementary Table S1). Agrobacterium tumefaciens-mediated transformation (strain EHA105) of embryonic calli was conducted, which was then transformed into Suijing18. Hygromycin phosphotransferase (hpt), as a plant-selectable agent, was used for screening rice-positive transformants.

### 4.4. Microscopical Observation of Internode Transverse Section

The individual internodes of the culm from the root to the top were in turn marked as LOI-I, LOI-II, LOI-III, and LOI-IV, which were, respectively, sampled at 14 days after heading and gently excised to keep them intact. Then, tissue samples were dehydrated, embedded in the accessory membrane, and sectioned after being fixed in a 70% FAA stationary solution (70% ethanol/35% formalin/acetic acid, 18:1:1) for two days [44]. Finally, a 0.05% crystal violet solution was used for staining. The paraffin section of the internode cross-section was observed with a light microscope (Olympus SZ61TR, Olympus, Tokyo, Japan).

### 4.5. RNA-seq

Shoot apex of mutant *rlck191* and Suijing18 (wild type) were sampled at the reproductive stage, the early stage of rice panicle differentiation, and three repetitions were derived from different plots. Sample tissues, which were collected to freeze immediately in liquid nitrogen, were bulked from two or three individuals for each repetition. RNA purification, reverse transcription, library construction, and sequencing were performed at Shanghai Majorbio Bio-Pharm Biotechnology Co., Ltd. (Shanghai, China) according to the manufacturer’s instructions. The RNA-seq transcriptome library was prepared following Illumina^®^ Stranded mRNA Prep, Ligation (San Diego, CA, USA). The sequencing library was performed on the NovaSeq X Plus platform (PE150) using the NovaSeq Reagent Kit.

### 4.6. Differential Expression Analysis and Functional Enrichment

To identify DEGs (differential expression genes) between two different samples, the expression level of each transcript was calculated according to the transcripts per million reads (TPM) method. RSEM [45] was used to quantify gene abundances. Essentially, differential expression analysis was performed using DEGseq [46]. DEGs with |log2FC| ≧ 1 and an FDR < 0.05 (DESeq2) or FDR < 0.001 (DEGseq) were considered to be significantly different expressed genes. In addition, functional enrichment analysis including GO and KEGG were performed to identify which DEGs were significantly enriched in GO terms and metabolic pathways at a Bonferroni-corrected *p* value < 0.05 compared with the whole transcriptome background. GO functional enrichment and KEGG pathway analysis were carried out by Goatools 1.4.8 and Python scipy software 1.14.1, respectively.

### 4.7. Assay of Quantitative Real-Time PCR

To verify the accuracy of RNA-seq data, the transcriptional levels of the DEG representative genes were detected using RNA used for RNA-seq in the wild type and mutant by qRT-PCR. Pre-treatment with 4×gDNA wiper Mix (Vazyme Biotech, Nanjing, China) ensured that the RNA template was completely free of residual genomic DNA, which guaranteed more reliable quantitative results. First-strand cDNA was prepared with 2 μg total RNA using Oligo (dT)20 primer and HiScript^®^ II Q RT SuperMix (Vazyme Biotech). qRT-PCR was performed using ChamQTM Universal SYBR^®^ qPCR Master Mix (Vazyme Biotech, Nanjing, China) on the LightCycler480 System (Roche, Basel, Switzerland). The PCR reaction mixture was adjusted according to the manufacturer’s instructions, and thermal cycling conditions consisted of 30 s at 95 °C, 40 cycles of 10 s at 95 °C, and 30 s at 61 °C. The specificity of PCR amplification was confirmed by a melting curve analysis for 15 s at 95 °C, 60 s at 60 °C, and 15 s at 95 °C. Gene expression in rice was normalized to OsUBQ (Table S1) as 2^−ΔΔCt^, where Ct is the cycle threshold measured according to a previous method. Each experiment was repeated for three biological replicates and three repeats of each replicate.

## 5. Conclusions

This study identified *OsRLCK191*, associated with culm strength and improving lodging resistance. Through the CRISPR/Cas9 method, a mutant of *OsRLCK191* was constructed for RNA-seq analysis to further reveal genes and proteins related to the biosynthesis pathways of culm strength that were significantly differentially expressed. The present study confirmed known culm strength-associated genes responding to the cytokinin oxidase/dehydrogenase family, cellulose synthase catalytic subunit genes, ethylene response factor family transcription factor, and squamosa promoter binding protein-like.

## Data Availability

The raw data supporting the conclusions of this article will be made available by the authors on request.

## Supplementary Materials

The following supporting information can be downloaded at: https://www.mdpi.com/article/10.3390/ijms252212382/s1.
