# Supplementary material for: A Novel Gene, OsRLCK191, Involved in Culm Strength Improving Lodging Resistance in Rice
Source: Int J Mol Sci. 2024 Nov 18;25(22):12382. doi: 10.3390/ijms252212382 (PMC11594765; doi:10.3390/ijms252212382)
Supplement: Supplementary file 1 [file ijms-25-12382-s001.zip › ijms-3239294-supplementary.pdf]

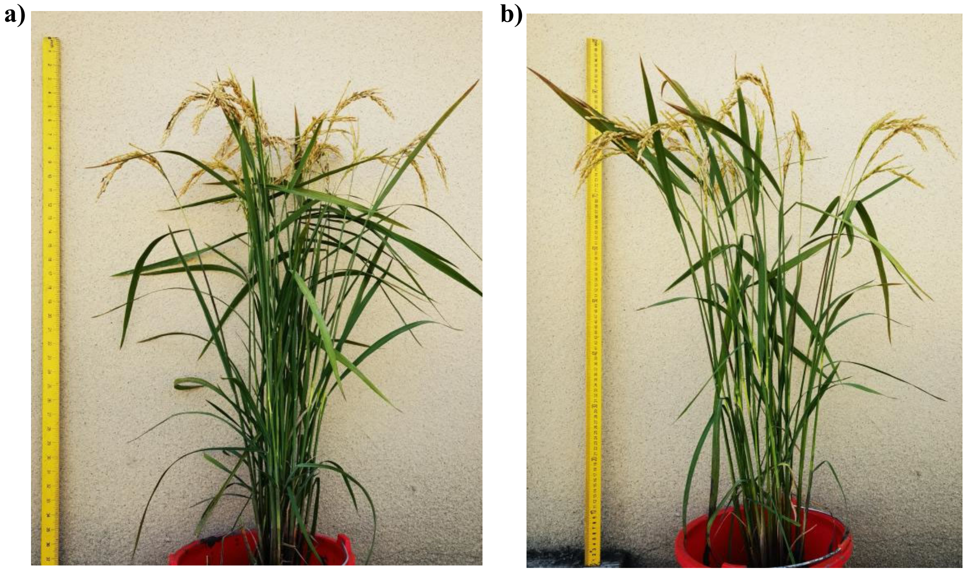

Figure S1. Gross morphology of plants. (**a**) Wild type Suijing18. (**b**) Mutant *rlck191*.

Table S1 Nucleotide sequence and corresponding primer sequences used in this study.

Primer name	Primer sequence	Usage
rlck191-sgRNA-F	TCGGACATGGCGGTGGTCTCgttttagagctagaaat	Sequence of sgRNA cassette
rlck191-sgRNA-R	GAGACCACCGCCATGTCCGAcggcagccaagccagca	Sequence of sgRNA cassette
rlck191-target	TCGGACATGGCGGTGGTCTC	Sequence of target site
rlck191-F	CCTCCCTGACCCCATTCAAC	Identification of the positive CRISPR plant
rlck191-R	TGAGTCAACACGCTCCTGTG	Identification of the positive CRISPR plant
Os01g0382400-F	TATGCAGTACCATCGCCTGT	qRT-PCR for *OsPR1#012*
Os01g0382400-R	GGATGCCTCCATCTCTGCTT	qRT-PCR for *OsPR1#012*
Os01g0797600-F	GGGTGCCAAGGCAAAAACAA	qRT-PCR for *ERF3*
Os01g0797600-R	CGCGGTGGAACAAATCCAAG	qRT-PCR for *ERF3*
Os04g0493400-F	CTACAAGGACTACTGCCGCC	qRT-PCR for *Cht4*
Os04g0493400-R	ACGTCACTTAGCTTCCAGCA	qRT-PCR for *Cht4*
Os05g0497300-F	CGTTGGCGAAGAAAGCCATC	qRT-PCR for *OsERF#074*
Os05g0497300-R	ATCCCGCTCCATAACAACGC	qRT-PCR for *OsERF#07*4
Os07g0685700-F	AGAGAGTCAGAGAGAGGTGGA	qRT-PCR for *EIL2*
Os07g0685700-R	TCCTCGATCGTAGCACAGAC	qRT-PCR for *EIL2*
Os11g0592200-F	TTCTCAAAGTGATGGCGGGG	qRT-PCR for *OsPR4*
Os11g0592200-R	CCGGTTCTTCACCTGGATAC	qRT-PCR for *OsPR4*
Os04g0577700-F	GCCTGTAGTAACCCCACACA	qRT-PCR for *OsOFP11*
Os04g0577700-R	CGTGATCAGATGAGGCCGAG	qRT-PCR for *OsOFP11*
Os03g0815100-F	GCGGGATCGCAAGTATCCTA	qRT-PCR for *SNAC1*
Os03g0815100-R	ACCCAATCATCCAACCTGAGA	qRT-PCR for *SNAC1*
Os01g0805300-F	GTTCGTGTTCTTCCGGGCTA	qRT-PCR for *PsbP*
Os01g0805300-R	GCAGAATGAACTCGTAAGGTTG	qRT-PCR for *PsbP*
Os01g0699500-F	ATTCCAAGGACCAAGAAATCGA	qRT-PCR for *MAP3K6*
Os01g0699500-R	GAACTCGAGGAACAGCTGGTA	qRT-PCR for *MAP3K6*

Os01g0704100-F	AATTGATGGTACGTGTCGTTTG	qRT-PCR for *NRT2.3*
Os01g0704100-R	CCACAGAAGTAAATCAAACCCG	qRT-PCR for *NRT2.3*
Os06g0493600-F	AACAAGGTGAACCGCTTCTA	qRT-PCR for *Pho1*
Os06g0493600-R	ATGTCTTTGGTGAGCTTTTCCT	*Pho1* qRT-PCR for
Ubiquitin-qRT-F	TGTGAAGATTGTGATGCCCTAC	qRT-PCR
Ubiquitin-qRT-R	CCACGCCAACGGATAAAA	qRT-PCR
